# Sero-Epidemiology of Foot and Mouth Disease in Domestic Ruminants in Amhara Region, Ethiopia

**DOI:** 10.3389/fvets.2019.00130

**Published:** 2019-04-30

**Authors:** Mekedes Mesfine, Seleshe Nigatu, Negus Belayneh, Wudu T. Jemberu

**Affiliations:** ^1^College of Veterinary Medicine and Animal Science, University of Gondar, Gondar, Ethiopia; ^2^Srinka Agricultural Research Center, Woldia, Ethiopia

**Keywords:** Amhara, epidemiology, foot and mouth disease, seroprevalence, risk factors, ruminants

## Abstract

This study involved cross-sectional serological and questionnaire-based surveys to investigate the sero-epidemiology of foot and mouth disease (FMD) in domestic ruminants, and farmers' knowledge and practices about the disease in the Amhara region of Ethiopia. A multistage cluster sampling was carried out to select domestic ruminants for serological sampling and for the interview with farmers. A total of 1,672 sera samples were collected and tested using a 3ABC-Enzyme Linked Immunosorbent Assay, and 170 farmers were interviewed. An overall FMD apparent seroprevalence of 11.48% (95% CI: 7.52–17.14%) was recorded in the domestic ruminants. The overall true prevalence was 12.04%. The seroprevalence of FMD was higher in cattle (14.37%) than in goats (7.10%) and sheep (7.07%). The age stratified seroprevalence in the districts showed that 66.67% of the districts studied experienced a FMD outbreak within the preceding year of the study time. A mixed effect logistic regression analysis revealed that agroecology, the production system and the age of the animal was significantly associated with FMD seropositivity in cattle (*P* < 0.05). A statistically significant (*P* < 0.05) positive correlation (*r* = 0.93) was observed between cattle and small ruminant FMD seroprevalences. About 82% of the farmers interviewed knew of FMD and 85% of them had experienced the disease in their own herds before. The farmers mostly employ traditional means to control FMD. In conclusion, the findings of the study indicated that FMD is a prevalent disease in the Amhara region with more importance in the intensive production systems and the lowlands of the region. High correlation in seroprevalence between small and large ruminants indicated a possible cross transmission between these species. Therefore, small ruminants should not be overlooked in FMD control. Farmers in the region have a good level of knowledge about the disease; however, currently they heavily rely on traditional practices primarily focused on treating wounds of infected animals. This calls for extension work on available effective preventive measures of the disease, such as vaccination and movement restriction.

## Introduction

Ethiopia possesses the largest domestic ruminants population in Africa, with an estimated population of about 60 million cattle, 30 million sheep, and 30 million goats (1). Despite this large resource base, the benefit derived from the livestock sector in Ethiopia is relatively low. Livestock diseases are among the many constraints which hinder the proper harnessing of the resource for food security and national development.

Foot and mouth disease (FMD) is one of the most important and highest priority livestock diseases globally (2). It's annual economic impact in terms of visible production losses and vaccination costs in endemic regions of the world is estimated between US$6.5 and 21 billion, whereas outbreaks in FMD free countries and zones cause losses of more than US$1.5 billion a year (3). Foot and mouth disease is a highly contagious disease in cloven-hoofed animals caused by the FMD virus (FMDV), a member of the *Aphthovirus* genus of genus the *Picornaviridae* genus. The FMDV has seven antigenically different groups of serotypes (O, A, C, Asia 1, SAT 1, SAT 2, and SAT 3) (4) which do not cross protect each other immunologically.

FMD is also among the most important livestock diseases that affects production and trade of animal and animal products in Ethiopia (5). Serosurveys in different parts of Ethiopia reported FMD with different degrees of prevalence reaching up to 26% (6–9). Outbreak incidence studies have also indicated that FMD occurs throughout the country with significant variation in geography and production systems (10). Among the seven serotypes of FMDV, four of them (O, A, SAT 2, and SAT 1) have been reported in Ethiopia in recent times (10, 11).

A sound knowledge of the epidemiology of the disease is required to apply effective control measures locally or nationally. Despite some seroprevalence studies mainly focusing on cattle in some parts of Ethiopia, a comprehensive epidemiological study of FMD in Amhara National Regional State (in short Amhara region) is not available. This study was aimed at investigating the seroepidemiology of FMD and assessing farmers' knowledge and practices about the disease in the Amhara region. The specific objectives were to determine the seroprevalence and to estimate the annual outbreak incidence of FMD in domestic ruminants, to identify factors associated with its occurrence, and to assess the knowledge and practice of farmers about the disease.

## Materials and Methods

### Study Area

**Figure 1:**
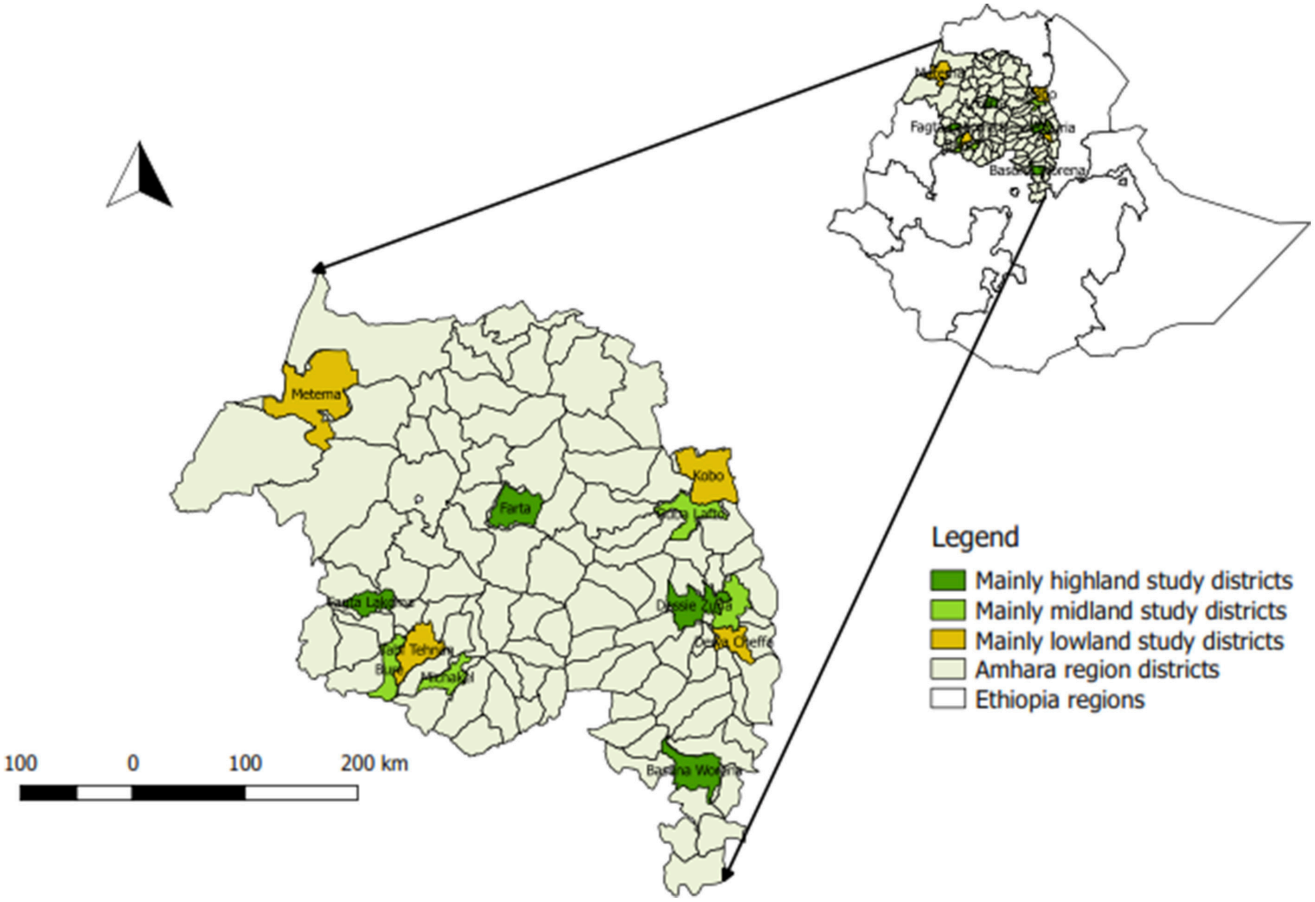
The map of Amahra region showing the study districts.

The study was conducted in the Amhara region, in northern Ethiopia, extending from 9° to 13.75°N latitude and 36° to 40.5°E longitude. The region is administratively divided into eleven zones and 113 districts (Figure 1). The region has a ruminant population of 15.45 million cattle, 9.79 million sheep and 6.08 million goats (1). Agro-ecological zones of the region are classified as lowlands (Tropical zone) with below 1,830 m altitude, 27°C average temperature and 510 mm annual rainfalls, midland (Subtropical zone) with 1,830–2,440 m altitude, 22°C average temperature and 510–1,530 mm annual rainfalls and highland (Cool zone) with above 2,440 m altitude, 16°C average temperature 1,530–2,000 mm annual rainfalls (12). The livestock production systems in the region is dominantly a subsistence crop-livestock mixed system.

### Study Population, Sampling Strategy, and Sample Size

The study was a cross-sectional study, which targeted the domestic ruminant population of the Amhara region. Individual cattle, sheep and goats were sampled for the study. A multistage cluster sampling was employed to select sampling units. The ruminant population of the study area can be structured as individual animals clustered into household herds, household herds into kebeles, kebeles into districts and then finally, district populations together constitute the regions ruminant population. First, the 12 districts that represent the three agro-ecological and two geographic zones of the region (western Amhara and eastern Amhara) were selected purposively. Among the districts within the same agro-ecology and geographical zone, the study districts were selected conveniently by their accessibility. Three kebeles that were considered typical of the district's agro-ecology in each selected district, were again selected purposively, based on the convenience of accessibility. Finally, as the animals from households within kebeles mix in common grazing areas and watering points, study animals from the selected kebeles were selected haphazardly to mimic random sampling. Strict random sampling was not possible due to difficulty in establishing a sampling frame in that traditional extensive production system.

The sample size was determined by the formula for the estimation of prevalence using random sampling as provided in Thrusfield (13). Substituting expected prevalence of 16% for cattle (14) and 5% for sheep and goat (15), a 95% level of confidence and a ±5% desired level of precision in the formula; sample sizes of 206 cattle, 72 sheep, and 72 goats were determined for the study. The sample sizes determined for the simple random sampling method above were adjusted for the cluster sampling method employed in the study, by multiplying it with the design effect of five giving sample sizes of 1,030 cattle, and 360 goats and 360 sheep. A large design effect of five was used because of the high clustering effect of FMD, due its high transmissibility (13, 16). The sample size was equally distributed across the 36 selected kebeles resulting in 30 cattle, 10 sheep, and 10 goats per kebele.

For the questionnaire survey of farmers that was conducted parallel to the serosurvey, five households were haphazardly selected from each of the 36 kebeles selected for the serosurvey, resulting in a total of 170 farmers.

### Sample and Data Collection

#### Serum Sample Collection

Blood samples were collected from the jugular vein of individual animals, using sterile plain vacutainer tubes. About six milliliters of blood was withdrawn from each animal. The samples were coded, stored overnight at room temperature for serum separation and transferred into sterile cryovials after separation. The sera samples were stored at −20°C until laboratory test was performed.

#### Data Collection

For each sampled animal biodata such as breed, age, sex, and husbandry data, such as production system, history of vaccination, history of clinical FMD infection and life history of the animal were collected to assess their association with FMD seropositivity. The breed of animal was categorized as exotic, cross and local. Age was categorized as young (<3 years of age for cattle and <18 months of age for small ruminants and adults >3 years of age for cattle and >18 months for small ruminants). Animals under 1 year of age were also noted separately for the sake of examining recent infection. The production system under which the sampled animals were kept was categorized as extensive livestock production systems (when animals are kept free-range for part or all of their production cycle), intensive livestock production systems (when animals are housed and hand fed) and semi intensive livestock production systems (when animals share the characteristics of both intensive and extensive production systems). The history of FMD infection and vaccination were recorded as “yes” or “no,” and the life history of the animal was categorized either as born within the herd, or as acquired from outside the herd. Later in the analysis, breed was collapsed into two categories as the number of animals found in the exotic category were very few and were lumped to the cross breed category, and the production system was similarly collapsed into two categories, as the number of animals found in the intensive category were very few and were lumped to semi intensive category.

#### Laboratory Diagnosis

The collected serum samples were subjected to FMD NSP Competition 3ABC ELISA (ID Screen®, ID.Vet, Montpellier,-France) at the National Animal Health Diagnostic and Investigation Centre, Sebeta, Ethiopia. The diagnostic test has a sensitivity of 91.7% and specificity of 99.5% (17). This test detects only antibodies produced against non-structural proteins and can hence differentiate vaccinated animals from unvaccinated animals. The tests were done according to the manufacturers recommendation and the procedure provided by the OIE manual of diagnostic tests and vaccines for terrestrial animals (18).

#### Questionnaire Survey

A semi structured questionnaire was prepared (Annex 1) mainly to assess the farmers' knowledge about FMD and their control practices, which was administered through interview. Knowledge was ascertained as “yes” (know of FMD) and “no” (do not know of FMD) by asking the farmers to describe the clinical and epidemiological features of the disease, and, if they were able to provide features of the disease that were consistent with a pre-specified case definition of the disease given in the questionnaire, they were considered to know of the disease and continued with the remaining interview. Their perception of impact was assessed on how serious the disease is in causing mortality. For the control practices, they were asked about all types of measures they take to tackle FMD before it occurs and during an outbreak. The questionnaire was first pilot tested in 10 farmers that were not included in the study and was revised for clarity to ensure the intended data would be collected.

The study protocol for the interview was ethically reviewed and approved by the Institutional Review Board (IRB) of the University Gondar. Oral informed consent was obtained from each farmer participating in the interview after reading a written consent form. The use of oral consent was approved by the IRB, considering the fact that most study participants could not read and write to provide their consent in writing. The consent form mainly explains the purpose of the study, the risks and benefits of participation in the study, conditions of confidentiality, and the right to refusal or withdrawal from the study at any time. The interviewers confirmed the participants' oral consent by signing on the respective consent form for each interviewee as per the Board's guidelines.

### Data Management and Analysis

Data generated from laboratory investigations were recorded and coded using a Microsoft Excel spreadsheet and analyzed using STATA version 14. Descriptive analyses were used to assess the seroprevalence, and knowledge and control practices of farmers. The seroprevalences were calculated as a proportion of seropositive to the total number of animals tested, and 95% confidence intervals were given for the estimates. Standard errors for the seroprevalence confidence intervals were calculated using the cluster sandwich variance estimator which accounts for the cluster correlated nature of the data (19). The true prevalence (TP) was derived from the apparent prevalence (AP) based on the sensitivity (Se) and specificity (Sp) of the diagnostic test as: TP = $\frac{AP+Sp-1}{Se+Sp-1}$ (13). Age stratified seroprevalence were used to estimate the annual occurrence of FMD outbreak. Seroprevalence difference between cattle and small ruminants was compared using the chi-squared test.

Mixed effect logistic regressions were employed to identify factors associated with the FMD seropositivity. In the mixed logistic regression, seropositivity status was the dependent variable and the putative FMD risk factors were the predictor variables. Districts and kebele were included as random effect predictor variables, to account for clustering that could arise from cluster sampling used in the data collection, and the putative risk factors were treated as fixed effect predictor variables. Separate analyses were performed for cattle and small ruminants. The predictor variables that were included in the models were FMD putative risk factors, such as species (only for the small ruminants model), age, sex, breed, FMD vaccination history, life history, agroecology and production system. First collinearity among the predictor variables was checked by correlation matrix and none of them were found collinear. Then the full models containing all predictors were run and statistically non-significant (*p*-value <5%) predictors were removed and the models run again. When removal of a predictor changed the coefficients of the remaining predictors by more than 30%, it was considered as a confounder and retained in the model (20). The models were run again in the same manner until only statistically significant predictors and confounders were left, resulting in the final models. Intraclass correlations (ICC's) were provided for random effect variables (district and kebele) using the *estat icc* post-estimation command of stata. These ICC's were calculated as the proportion of variances at a given cluster level (random effect variable) to the total variance (sum of cluster variances and residual variance) conditional on the effect of the fixed effect predictors (20).

The association between seropositivity and the clinical FMD infection history of animals in the preceding 3 years, was tested to evaluate the strength of recall of farmers with regard to FMD infection events in their herd. The Pearson correlation coefficient was used to measure the strength of correlation between cattle and small ruminant seroprevalence at kebele level. In all the analyses, the confidence level was held at 95% and *P* < 0.05 was set for statistical significance. Geographic distribution of the disease in the study area was mapped using GIS software QGIS version 2.18.

## Results

### Seroprevalence

A total of 1,750 serum samples; 1,030 from cattle, 360 from sheep, and 360 from goats were collected for the study and 1,672 of these samples were tested for a FMDV antibody. The remaining 21 samples from cattle, eight samples from sheep and four samples from goats did not fit the test and were therefore discarded. Forty-five ovine serums were not tested due to a shortage of laboratory test kits.

**Figure 2:**
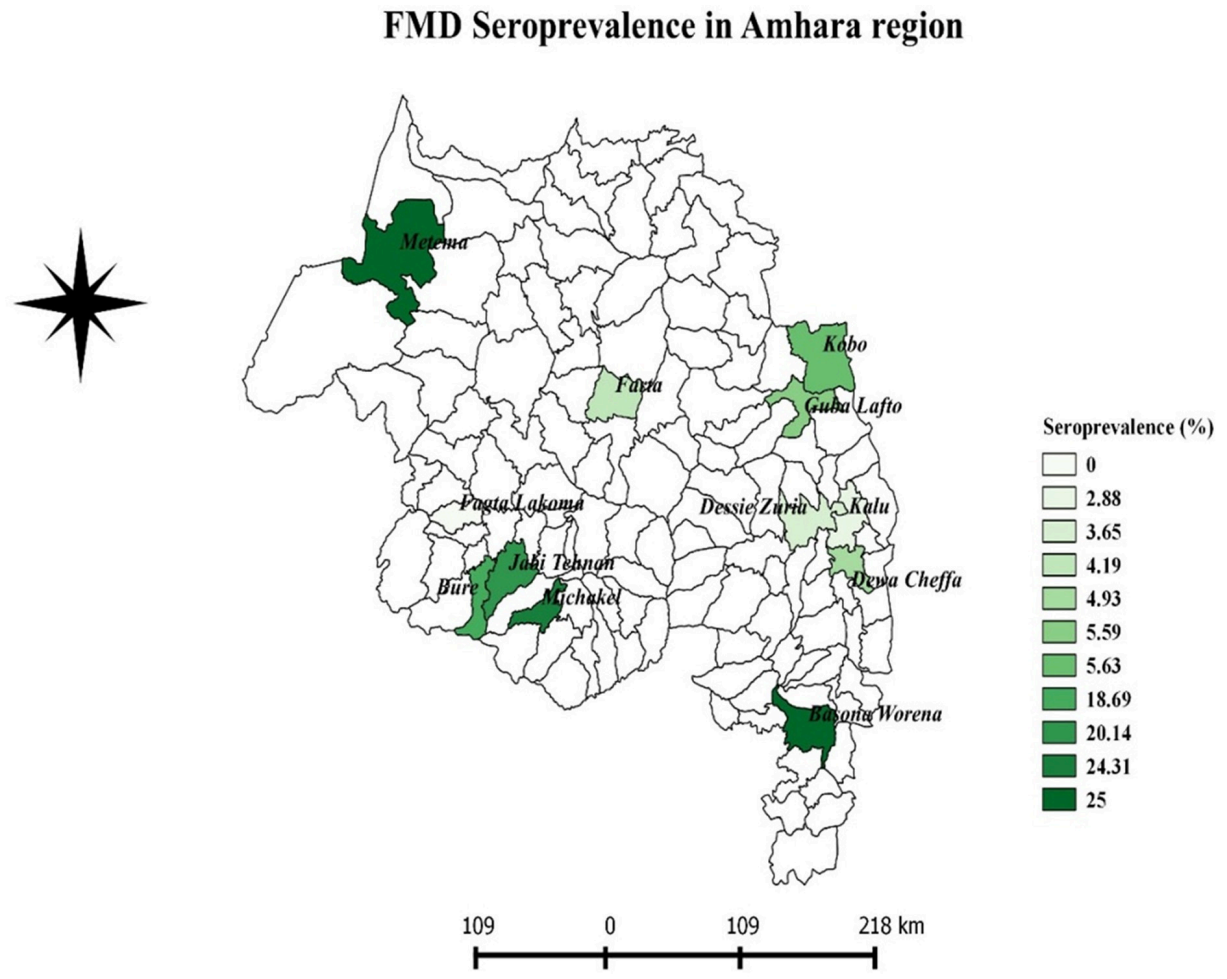
The geographic distribution of FMD seroprevalence in Amhara region.

A FMD antibody was detected in domestic ruminants of 11 districts, out of the total 12 sampled districts in the Amhara region (Table 1). The overall apparent seroprevalence of FMDV in the domestic ruminants in the region was 11.48% (95% CI: 7.52–17.14%). The overall true prevalence adjusted for the sensitivity and specificity of the imperfect diagnostic test used was 12.04%. The seroprevalence of FMD in cattle (14.37%) was statistically significantly higher than that of goats (7.10%) and sheep (7.07%) (*P* < 0.001). The seroprevalence was variable among the study districts. The highest seroprevalence was observed in Metema and Basonaworana districts (25%) and the lowest was in Fagta Lakoma district with no seropositive animals detected. Geographically, districts in the western Amhara region had relatively higher seroprevalence compared to districts in the eastern Amhara region (Figure 2). The age stratified seroprevalence in the districts showed that 66.67% (8/12 districts) of the study districts had FMD seropositive ruminants that were ≤ 1 year of age (Table 2).

**Table 1:** Seroprevalence distributions of FMD in domestic ruminants in the study districts.

**District**	**Number of samples**	**Number of seropositive (Seroprevalence in %)**	**95% CI of the seroprevalence (%)**
Basonaworana	136	34 (25)	9.42–51.63
Dewa cheffa	142	7 (4.93)	3.40–7.10
Kalo	139	4 (2.88)	1.94–4.24
Dessie zuria	137	5 (3.65)	2.53–5.22
Kobo	142	8 (5.63)	3.21–9.70
Guba lafto	139	5 (3.59)	2.58–4.99
Jabi tehnan	144	29 (20.14)	11.68–32.47
Michakel	144	35 (24.31)	10.37–47.13
Bure	123	23 (18.69)	7.54–39.33
Fagta lakoma	139	0 (0)	–
Farta	143	6 (4.19)	1.12–14.53
Metema	144	36 (25)	10.39–50.66
Total	1,672	192 (11.48)	7.52–17.14

**Table 2:** Seroprevalence of FMD in domestic ruminants ≤1 year of age in the study districts.

**District**	**Number of samples**	**Number of seropositive (seroprevalence in %)**	**95% CI of Seroprevalence (%)**
Basonaworana	26	5 (19.23)	2.90–64.95
Dewa cheffa	39	1 (2.56)	0.58–10.64
Kalo	27	1 (3.70)	0.87–14.47
Dessia zuria	17	0	0
Kobo	22	0	0
Guba lafto	38	1 (2.63)	0.40–14.97
Jabi tehnan	33	4 (12.12)	4.50–28.65
Michakel	45	6 (13.33)	2.30–50.91
Bure	27	2 (7.41)	1.28–33.04
Fagta lakoma	27	0	0
Farta	34	2 (5.88)	0.90–30.01
Metema	25	0	0
Total	360	22 (6.11)	2.84–12.64

### Factors Associated With FMD Seropositivity

The association of putative risk factors, such as animal factors (age, sex, and breed), FMD vaccination history, life history of animals in the herds, agro-ecology and production systems were evaluated. The putative risk factors, such as age, agro-ecology, and production systems were statistically significantly associated with FMD seropositivity in cattle (*P* < 0.05) (Table 3). For small ruminants only the sex and production systems were statistically significantly associated with FMD seropositivity (*P* < 0.05) (Table 4).

**Table 3:** Factors associated with FMD seropositivity using mixed effect logistic regression analysis in cattle.

**Variables**		**Number sampled**	**Seroprevalence (%)**	**Odds ratio (95%CI)**	***P*-value**
Agro ecology	Lawland^*^	347	17.87	–	–
	Midland	330	14.55	0.17 (0.04–0.85)	0.031
	Highland	332	10.54	0.81 (0.20–3.25)	0.769
Age group	Adult^*^	614	18.57	–	–
	Young	395	7.85	0.37 (0.23–0.59)	<0.001
Production system	Extensive^*^	921	12.70	–	–
	Intensive and semi intensive	88	31.82	9.37 (2.36–37.25)	0.001

* *Reference category*.

*Intraclass correlation (ICC) for the random variable District = 0.12 (95% CI: 0.02–0.44) and for Kebele = 0.30 (95% CI: 0.17–0.48)*.

**Table 4:** Factors associated with FMD seropositivity using mixed effect logistic regression analysis in small ruminants.

**Variables**		**Number sampled**	**Seroprevalence (%)**	**Odds ratio (95%CI)**	***P*-value**
Sex	Female^*^	461	8.89	–	–
	Male	202	2.97	0.34 (0.13–0.86)	0.023
Production system	Extensive^*^	628	6.37	–	–
	Intensive and semi intensive	35	20	8.4 (1.12–62.76)	0.038

* *Reference category*.

*Intraclass correlation (ICC) for the random variable District = 2.07e-33 and for Kebele = 0.41(95% CI: 0.20–0.66)*.

History of FMD clinical infection was significantly associated with seropositivity in cattle (*P* < 0.05).

A strong positive correlation (*r* = 0.93) was observed between district level cattle and small ruminant's FMD seroprevalence. The correlation between the two seroprevalence was statistically significant (*p* < 0.05).

### Farmers' Knowledge About FMD and Their Control Practices

Of the 170 farmers interviewed, 82.4% of them knew of the disease and 85% of them had experienced the disease in their herd. Ninety six percent of the farmers mentioned that cattle are the livestock species most affected by FMD. The respondent farmers had experienced mortality due to FMD only in cattle. Most farmers responded that mortality due to FMD is more common in younger or male animals than in older or female animals. The majority of farmers responded that FMD occurs from August to December. About 78% farmers that responded about the source of FMD outbreak think that the main sources of FMD is herds mixing with infected animals at communal grazing and watering points, and about 22% of them think introduction of infected cattle from markets is the main source of FMD infection.

In the study areas there was no official FMD control. However, farmers used different control and prevention measures, before and during the occurrence of the disease. About 48% of farmers practiced one or more type of FMD control measure. The control methods practiced in the study area by percent of farmers practicing them included vaccination (3%), isolation and nursing (6%), antibiotic therapy for secondary complication (12%), and traditional methods (27%). The most commonly used traditional treatment methods were dressing the FMD lesion with *araki* (local liquor), honey, boiled cabbage, fumigation with herb smoke, salt and hot chilies.

## Discussion

### Seroprevalence and Factors Associated With FMD Seropositivity in Domestic Ruminants

In this study an FMDV antibody has been detected in domestic ruminants in 11 of the 12 districts sampled in the Amhara region, indicating the endemic nature of the disease in the region. The serosurvey revealed that FMD is a significant disease of domestic ruminants in the region with an overall prevalence of 11.48%. This generalization of the prevalence estimate to the region ruminant population should, however, be taken cautiously as the selection of ruminants for the survey was not strictly by random sampling.

The seroprevalence was higher in cattle (14.4%) than in sheep and goat (7.1%). The seroprevalence in cattle determined in this study was toward the middle of the seroprevalence range of 6% to 26% reported by previous studies in different parts of Ethiopia (6–8, 15, 21, 22). Most seroprevalences that are higher than determined in this study were reported in southern pastoral areas of Ethiopia (8, 23). This could be due to higher livestock mobility in the pastoral system, which facilities high contact and spread of the disease, as compared to the crop-livestock mixed system which is the main type of production system in the current study area.

Variation in spatial distributions of FMD seroprevalence have been observed across the region. The highest level of FMD seroprevalence was found mainly in the western Amhara districts. The presence of cattle trade routes to Sudan might be a factor for this observed higher FMD prevalence in western Amhara. This is further strengthened by the observation that Metama district, which borders Sudan and contains the port to Sudan, has the highest prevalence. Animal trade movement is known to be the main contributor of transmission of transboundary diseases (24).

The age stratified seroprevalence indicated about two thirds of sampled districts had FMD seropositive in animals of less or equal to 1 year of age. This particular data indicates the extent of recent FMD viral activity in the region's domestic ruminant population. This might be interpreted as about two thirds of districts in the region are likely to be affected by an FMD outbreak every year. The same herd in a district is unlikely to be infected every year, though this could be possible with different serotypes. But the most likely scenario could be that a part of the district is affected in one year and the other part another year, as districts are not single epidemiological units.

Statistically significant seroprevalence variation was observed in the different species of domestic ruminants. Cattle were almost twice more likely to be seropositive compared to sheep and goats. Beyene et al. (15) similarly reported a significantly higher prevalence of infection in cattle than in sheep and goat in western Ethiopia. This indicates that FMD is primarily transmitted among cattle in mixed species of ruminant production. In the crop-livestock mixed system, cattle move and intermingle more than small ruminants as they are used for agriculture purposes like plowing, threshing and land bed preparation, which might contribute to a higher transmission in cattle. This study found a statistically significant (*p* < 0.05) association between FMD seropositivity and the age group in cattle in which seroprevalence was higher in older animals. This finding is in line with the reports of Megersa et al. (6), Bayissa et al. (8), Jenbere et al. (22), Habtamu et al. (23), Yahya et al. (9), and Beyene et al. (15) who found statistically higher prevalence in older animals. This may be related to cumulative infection through time in which older animals have more chance to get infected during their longer stay in the population. In crop-mixed mixed production systems, which is the dominant production system in the Amhara region, the cattle offtake rates are low (25) and animal stay in the herds longer, therefore, cumulative seropositivity for disease should be common.

Agro-ecologically the study showed significantly higher seroprevalence in the lowland compared to the midland. This finding was in line with the reports of Megersa et al. (6) who reported higher seroprevalence of FMD in lowland areas compared to the highlands in southern Ethiopia. The higher prevalence of the disease in the lowlands could be due to the production system which allows for more animals to mix, compared to the midland.

Higher seroprevalence was seen in intensive and semi intensive production systems rather than extensive production systems. Similarly, a higher incidence of FMD outbreak was reported in more intensive production systems than extensive systems nationally by Jemberu et al. (10). This may due to a high rate of contact between animals within intensively managed herds, facilitating the transmission of infections.

Animals that had been reported to have a previous history of clinical FMD infection, were almost four times more likely to be seropositive compared to cattle that had no history of infection. This analysis seems trivial and the result obvious. But it provides important information about the farmers' ability to identify and recall previous FMD infection in their cattle herd, which has an advantage for the questionnaire-based study on FMD.

The current study reported a statistically significant (*p* < 0.05) association between FMD seropositivity and sex in small ruminants. Seroprevalence was higher in females than in males. Similarly, a higher FMD seroprevalence in females was reported in the Benjie Maji areas of southern Ethiopia (26). Females are kept for an extended duration of time as compared to males, for breeding, which might result in a higher seroprevalence of FMD in females.

In our preceding sections we reserved to discuss the factors found associated with FMD seropositivity in terms of risk factors. It is argued that in a cross-sectional study design it is difficult to speak of risk, as exposure and outcome are measured at the same time and risk is defined as the probability of an outcome in a population or the probability that a specific outcome or disease will develop during a specific period of time (27). Most of the time cross-sectional studies can only provide a hypothesis about risk factors.

The study revealed a strong positive correlation between district level cattle and small ruminant FMD seroprevalences. This might indicate cross species transmission of infection between cattle and small ruminants. With the available data, it was not possible to ascertain whether the cross-species transmission is bidirectional or unidirectional. However, given the observed higher prevalence in cattle (double that of sheep and goat), one can guess that transmission is higher in cattle and that sheep and goat probably get infection mainly by cattle.

### Farmers’ Perceptions About FMD and its Control Measures

Most of the farmers in the study areas were able to describe clinical pictures of FMD that were consistent with the given case definition of FMD. Most of them expressed that they had experienced the disease in their herds and further indicated that the disease is fatal for calves and is seasonal with the highest incidence from August to December. This indicates that the farmers know the disease and its impact very well. However, only few respondents practiced vaccination and animal movement control/herd isolation as a method of FMD control. Most use traditional practices/medicines mainly for the treatment of the sick animals, by dressing the wounds. The good level of knowledge the farmers have is important to initiate intervention, however, the fact that farmers rely on traditional practices focused only on treating the wounds of infected animals calls for educating them about the available effective preventive measures of the disease, such as vaccination, and herd isolation during an outbreak.

## Ethics Statement

The study was carried out in accordance with guidelines of animal ethics committee of College of Veterinary Medicine and Animal sciences, University of Gondar and the study protocol was approved by the committee.

## Author Contributions

MM collected and analyzed the data and wrote the manuscript. SN conceived and designed the study, and revised the manuscript. NB collected and analyzed the data. WJ conceived and designed the study, analyzed the data, and wrote the manuscript.

### Conflict of Interest Statement

The authors declare that the research was conducted in the absence of any commercial or financial relationships that could be construed as a potential conflict of interest.
