# Supplementary material for: Sero-Epidemiology of Foot and Mouth Disease in Domestic Ruminants in Amhara Region, Ethiopia
Source: Front Vet Sci. 2019 Apr 30;6:130. doi: 10.3389/fvets.2019.00130 (PMC6503644; doi:10.3389/fvets.2019.00130)
Supplement: Supplementary file 1 [file Data_Sheet_1.pdf]

Annex 1: Semi structured questionnaire to assess farmer's knowledge about FMD and their control practices

A. General information

1. Owner's Name __________sex ______ Zone ________District ___________kebele ______

2. Number of Livestock owned

Cattle_______sheep______goats______horse______donkey_____poultry_____others____Total ___

B. **Disease knowledge and impact information**

1. Do you Know FMD? (Consider yes and continue with the questionnaire if he/she mentions one of the typical features of the disease described in the following case definition : Lameness due to foot lesion and salivation due to mouth lesion in cattle and/ or small ruminants; Foot lesion in cattle and/ or small ruminants that is contagious and causing several morbidity in the herd; Mouth lesions in cattle and/ or small ruminants that is contagious and causing several morbidity in the herd; or Lameness or mouth lesions, and blisters (sores) on teats of cows.

   A. Yes B. No

2. Have you had the disease outbreak in your herd?

   A. Yes B. No.

3. If yes, what do you think the source of outbreak in your herd ____________________________.

4. Which species of ruminants are mostly affected by FMD? ________________________________

5. How much common is mortality due to FMD?

   A. low B. high

6. In which species of animals is mortality common?

   A. cattle B. small ruminants C. both

7. In which sex of animals is the mortality common

   A. Male b. Female

8. In which age group of animals is mortality common?

   A. young B. adult

9. When is the disease common in your locality (kebele)?

   Mention months________________

C. Disease control practice information

1. Is there any government organized FMD control in your locality (kebele)

   A. Yes B. No

2. Do you practice any FMD control measure for your herd?

   A. Yes B. No

3. If yes, list the control measures you apply in your animals________________

4. When are control measure implemented?

   A. Before the outbreak B. During the outbreak

5. Do you vaccinate your cattle against FMD?

   A. Yes B. No

6. Do you vaccinate small ruminants against FMD?

   A. Yes B. No

7. When do you vaccinate against FMD?

A. Before occurrence of the disease B. when disease outbreak occurrence
